# Evaluation of age, weaning weight, body condition score, and reproductive tract score in pre-selected beef heifers relative to reproductive potential

**DOI:** 10.1186/s40104-019-0329-6

**Published:** 2019-02-26

**Authors:** Sarah E. Dickinson, Michelle F. Elmore, Lisa Kriese-Anderson, Joshua B. Elmore, Bailey N. Walker, Paul W. Dyce, Soren P. Rodning, Fernando H. Biase

**Affiliations:** 1 Department of Animal Sciences, Auburn University, 559 Devall Dr, Auburn, AL 36839 USA; 2 Alabama Cooperative Extension System, Auburn, AL USA

**Keywords:** Fertility, Phenotypic selection, Pregnancy outcome, Replacement heifer

## Abstract

**Background:**

Artificial insemination is a preferred breeding method for beef heifers as it advances the genetic background, produces a predictive and profitable calving season, and extends the heifer’s reproductive life span. As reproductive efficiency in heifers is key for the success of beef cattle production systems, following artificial insemination, heifers are exposed to a bull for the remainder of the breeding season. Altogether, up to 95% of heifers might become pregnant in their first breeding season. Heifers that do not become pregnant at the end of the breeding season represent an irreparable economical loss. Additionally, heifers conceiving late in the breeding season to natural service, although acceptable, poses serious losses to producers. To minimize losses due to reproductive failure, different phenotypic parameters can be assessed and utilized as selection tools. Here, we tested the hypothesis that in a group of pre-selected heifers, records of weaning weight, age at weaning, age at artificial insemination, and age of dam differ among heifers of varied reproductive outcomes during the first breeding season.

**Results:**

None of the parameters tested presented predictive ability to discriminate the heifers based on the response variable (‘pregnant to artificial insemination’, ‘pregnant to natural service’, ‘not pregnant’). Heifers categorized with body condition score = 6 and reproductive tract score ≥ 4 had the greatest proportion of pregnancy to artificial insemination (49% and 44%, respectively). Furthermore, it was notable that heifers presenting body condition score = 6 and reproductive tract score = 5 presented the greatest pregnancy rate at end of the breeding season (89%). Heifers younger than 368 d at the start of the breeding season did not become pregnant to artificial insemination. Those young heifers had 12.5% chance to become pregnant in their first breeding season, compared to 87.5% if the heifers were older than 368 days.

**Conclusion:**

Our results suggest that beef heifers with body condition score = 6 and reproductive tract score ≥ 4 are more likely to become pregnant to artificial insemination. Careful assessment should be undertaken when developing replacement heifers that will not reach 12 months of age by the beginning of the breeding season.

**Electronic supplementary material:**

The online version of this article (10.1186/s40104-019-0329-6) contains supplementary material, which is available to authorized users.

## Background

Reproductive inefficiency is a limiting factor in beef cattle production systems. In females, reproductive failure is assumed when animals do not become pregnant within the breeding season or conceive but do not maintain pregnancy to calving [1]. In beef heifers, pregnancy rates range from 53% to 95% under natural service [2–10] and are reduced to 48% to 69% when artificial insemination (AI) is the only breeding strategy utilized [2–9, 11, 12]. The negative impacts of reduced pregnancy rates in beef heifers contribute to the overall production deficit of the cattle operation that cannot be recovered in the following years [13]. Therefore, the selection and management of replacement heifers to obtain greater reproductive success within their first breeding season is of great importance to beef cattle production systems [14].

Many management practices aim at maximizing the percentage of heifers pregnant at the end of the breeding season by increasing the percentage of pubertal heifers entering the breeding season. Most strategies include the selection of heifers that reach appropriate age and 55–65% of the projected mature body weight before the start of the breeding season [10, 15]. Additionally, heifers can be selected based on reproductive tract [9, 16] and body condition scores (RTS and BCS, respectively) prior to breeding. Heifers that do not meet these criteria are usually considered poor replacement candidates. Furthermore, producers can implement a progestin based estrous synchronization protocol [9, 10] for induction of cyclicity in peripubertal heifers.

Genetic selection has been used extensively to improve production traits in beef cattle, however there are challenges to using genetic selection to improve reproduction. First service conception and pregnancy rate are used to indicate fertility in heifers. However, unlike growth and carcass traits, the heritability of female reproductive traits is low, for example, 0.03–0.18 [2, 5, 17] and 0.02–0.13 [2, 5, 18] for first service conception and pregnancy rate, respectively. The low heritability and polygenic nature of fertility traits make it difficult to utilize statistical models to select animals to improve heifer fertility.

While limited improvement in female fertility can be made through genetic selection, the implementation of appropriate management practices does increase the likelihood of reproductive success in heifers. However, even when the aforementioned management practices are followed, a percentage of beef heifers still fail to become pregnant or breed later into the breeding season. Therefore, we aimed to determine if phenotypic parameters differed among heifers of varied pregnancy outcomes. We tested the hypothesis that in a group of heifers managed according to best management practices, records of weaning weight, age at weaning, age at artificial insemination, and age of dam would differ among heifers of varied reproductive outcomes during the first breeding season.

## Methods

All animals sourced in this study belonged to Auburn University. All procedures with animals were performed in accordance with the protocols approved by Institutional Animal and Care and Use Committee in Auburn University.

### Overall nutritional management of heifers

The dataset used in this study contained the first breeding season pregnancy outcome, phenotypic, and pedigree records for crossbred, beef heifers (Angus × Simmental cross; *n* = 259) born in the years 2010 to 2016 at three Auburn University Experimental Stations (Black Belt Research and Extension Center (*n* = 53); Gulf Coast Research and Extension Center (*n* = 136); Wiregrass Research and Extension Center (*n* = 70)). At weaning, a proportion of heifers born each year was retained as potential replacement heifers.

Heifers were managed to reach a target weight of 60% of their mature bodyweight (approximately 381 kg) by the start of their first breeding season, which began in early December of each year. Heifers at the Black Belt Research and Extension Center were weaned and developed on toxic endophyte infected tall fescue pastures and free-choice ryegrass hay. Heifers were supplemented as needed with a 50:50 mixture of corn gluten pellets and soyhull pellets. Heifers remained on tall fescue pastures from weaning through the winter grazing season and the time of pregnancy diagnosis. Heifers at the Gulf Coast Research and Extension Center were developed from weaning to breeding on bahiagrass pasture alongside free choice ryegrass hay. Heifers were supplemented as needed with a Nutrena NutreBeef® 13% protein pellet. After breeding, heifers were moved to a ryegrass pasture for the remainder of the winter grazing season. At the Wiregrass Research and Extension Center, weaned heifers were managed on bermudagrass pasture with supplementation of 50% pelleted soyhulls and 50% corn gluten feed that was provided as needed. As summer pastures entered dormancy, heifers were fed free-choice Tifton-85 bermudagrass hay and were allowed to graze pastures containing triticale, hairy vetch, and rape seed for the remainder of the winter grazing season.

### Classification of heifers based on reproductive outcome

Approximately 30 d before the start of their first breeding season, heifers were evaluated for BCS (scale of 1–9 with 1 = emaciated and 9 = obese; [19, 20]) and assessed for RTS (scale of 1–5; 1 = pre-pubertal, 5 = pubertal, luteal phase; [21]) by a single, experienced veterinarian. At approximately 14 months of age (418.7 ± 22.6 days), heifers retained as replacements underwent estrous synchronization for fixed-time artificial insemination utilizing the 7-Day Co-Synch + CIDR protocol [22] to begin their first breeding season. Briefly, heifers received an injection of GnRH (i.m.; 100 μg; Cystorelin®; Merial, Duluth, GA) and insertion of a CIDR (intravaginal insert; 1.38 g progesterone; Eazi-Breed® CIDR®; Zoetis Inc., Kalamazoo, MI) on d −9, followed by CIDR removal and an injection of prostaglandin F2α (PGF; i.m.; 25 mg; Lutalyse®; Zoetis Inc., Kalamazoo, MI) on d −2. All heifers then received a second GnRH injection (i.m.; 100 μg; Cystorelin®; Merial, Duluth, GA) and were inseminated with a dose of semen of proven fertility on d 0, 54 ± 2 h after CIDR removal and PGF injection. Two professionals in random rotation were responsible for AI procedures at each experimental station for each year.

Fourteen days after insemination, heifers were exposed to two fertile bulls for natural breeding for the remainder of the breeding season. An experienced veterinarian performed initial pregnancy evaluation by transrectal palpation on d 62–89 post insemination, followed by final pregnancy evaluation on d 85–176 post insemination. Presence or absence of a conceptus, alongside morphological features indicating fetal age were recorded, and heifers were classified as “pregnant to AI’ (Preg AI), ‘pregnant to natural service’ (Preg NS), or ‘not pregnant’ (Not Preg).

### Phenotypic dataset

All analytical procedures were carried out in R software [23]. A schematic diagram representing the phenotypic data, the reproductive data, the merging, and the curation procedures is depicted in (Additional file 1: Figure S1). We obtained performance records and pedigree information for all calves born at each station from 2000 to 2017 from the Alabama Beef Cattle Improvement Association (*n =* 2530). We then filtered this dataset to include only heifer calves (*n* = 1240) and merged the performance dataset with records for pre-breeding body condition score, reproductive tract score, artificial insemination date, and pregnancy outcome for all heifers exposed to breeding during their first breeding season. We computed age of dam by subtracting the year of birth for the dam from the year of birth for each heifer.

We curated the data and eliminated observations that appeared as abnormal data imputation or outliers. We retained records if weaning weight was recorded within 158.8–453.6 kg and adjusted weaning weight was less than 453.6 kg. For analyses of pregnancy outcome, we only retained records for heifers that conceived if the pregnancy was carried out to term and a healthy calf was born. Heifers experiencing pregnancy loss (*n* = 3) were removed from the dataset because conceptus losses were not the focus of this study and analyzing data from these heifers would create a confounding category between pregnant and not pregnant. In addition, heifers presenting RTS < 3 (*n* = 5) were removed from the dataset according to consistent data supporting the notion that heifers with an immature reproductive tract are significantly less likely to become pregnant [9, 16].

We assessed normality of the continuous traits by performing a Shapiro-Wilk test and by examining histograms, quantile-quantile, and density plots for each parameter. We utilized the data from all heifers to assess the normality of weaning weight, adjusted weaning weight, and age at weaning, regardless of whether we collected breeding data. We assessed normality of age at AI using the data from the heifers that were artificially inseminated. Amongst heifers included in this dataset, the variables weaning weight (WW) and adjusted weaning weight (adj WW) were normally distributed (*P* > 0.01, Shapiro-Wilk test, Additional file 1: Figure S2). The variables age at weaning and age at AI displayed a deviation when tested from normal distribution (*P* < 0.01, Shapiro-Wilk test, Additional file 1: Figure S2). Nonetheless, visual inspection of the data (Additional file 1: Figure S2) indicated strong resemblance of normal distribution and the skewness was likely an influence of the varied management strategies at each station.

### Analysis of phenotypic parameters relative to pregnancy outcome

We analyzed the data using a mixed effect multinomial logistic regression model [24] because the heifers were categorized according to discrete reproductive outcomes. We modeled the phenotypic parameters relative to the reproductive outcomes according to two scenarios.

First, we accounted for the probability of three possible reproductive outcomes: Preg AI, Preg NS, or Not Preg. The variables station (S_*j*_, *j* = 1, 2, 3), AI year (Y_*k*_, *k* = 2011, 2012, 2013, 2015, 2016, 2017), BCS (BCS_l_, *l* = 4, 5, 6, 7), RTS (RTS_*m*_, *m* = 3, 4, 5), age at weaning (AgeW), age at AI (AgeAI), dam age (AgeD), and weaning weight (WW) were considered for the model. Heifer’s sires, nor the bulls used in the breeding programs were included in the model as they were confounded with stations. The probabilities (Pr) of occurrence of each pregnancy outcome relative to the variables were estimated as follows:

$$ \mathit{\ln}\left(\frac{\Pr (PregAI)}{\Pr (NotPreg)}\right)={\beta}_{01}+{\beta}_{11}{S}_j+{\beta}_{21}{Y}_k+{\beta}_{31}{BCS}_l+{\beta}_{41}{RTS}_m+{\beta}_{51} AgeAI+{\beta}_{61} AgeD+{\beta}_{71} WW+{\varepsilon}_1 $$

(and)

$$ \mathit{\ln}\left(\frac{\Pr (PregNS)}{\Pr (NotPreg)}\right)={\beta}_{02}+{\beta}_{12}{S}_j+{\beta}_{22}{Y}_k+{\beta}_{31}{BCS}_l+{\beta}_{42}{RTS}_m+{\beta}_{52} AgeAI+{\beta}_{62} AgeD+{\beta}_{72} WW+{\varepsilon}_2 $$

Next, we accounted for the probability of two outcomes only: ‘pregnant’ or ‘not pregnant’. The binomial modeling followed the same structure as presented above with exception that the dependent variable was represented by $\mathit{\ln}\left(\frac{\Pr (Preg)}{\Pr \left( Not\ preg\right)}\right)$.

We used the ‘nnet’ package [25] to fit the multinomial and binomial models. The likelihood of the ratios was calculated with a χ^2^ test using the ‘Anova’ function in the ‘car’ package. The model was assessed by the Akaike Information Criteria (AIC) [26] using the ‘MASS’ package. Statistical significance was inferred if *P* < 0.05.

The codes utilized for the analyses presented on this paper can be found on Additional file 2.

## Results

### Phenotypic description of beef heifers

The initial dataset contained performance data for 1240 heifer calves born on three Auburn University Experimental stations from 2000 to 2017 (Additional file 1: Figure S1). Following data filtering, 935 records indicated a weaning weight of 278.0 ± 35.5 kg, an adjusted weaning weight of 266.2 ± 30.4 kg, and an age at weaning of 227.2 ± 32.6 d for all heifers The 259 records obtained for heifers with pregnancy data demonstrated a weaning weight of 294.7 ± 38.9 kg, an adjusted weaning weight of 278.7 ± 26.6 kg, and an age at weaning of 229.2 ± 34.3 d (Table 1, Additional file 1: Figure S2). At the time of AI, the heifers included in our pregnancy outcome analysis averaged 418.7 ± 22.6 days of age (Table 1, Additional file 1: Figure S2).

**Table 1:** Descriptive statistics of continuous variables from beef heifers

Variable	Dataset	No. of records	Mean	SD	95% CI
Weaning weight, kg	All heifers^a^	935	278.0	35.5	275.8–280.3
	Pregnancy heifers^b^	259	294.7	38.9	289.9–299.4
Adj weaning weight, kg	All heifers	935	266.2	30.4	264.2–268.1
	Pregnancy heifers	259	278.7	26.6	275.5–282.0
Age at weaning, d	All heifers	935	227.2	32.6	225.1–229.3
	Pregnancy heifers	259	229.2	34.3	225.0–233.4
Age at AI, d	Pregnancy heifers	259	418.7	22.6	416.0–421.5

^a^All heifer calves recorded in the database from each station that were born between 2000 and 2017

^b^Heifer calves recorded in the database from each station that were subjected to AI between 2011 and 2017

*SD* Standard deviation, *CI* Confidence interval

All 259 heifers in the dataset analyzed for pregnancy outcome had a pre-breeding BCS of 4–7, with 81% of the heifers classified as 6 (Table 2, Additional file 1: Figure S3). The heifers were categorized between 3 and 5 for RTS, with 40 and 52% of the heifers presenting RTS 4 and 5, respectively (Table 2, Additional file 1: Figure S3). Altogether, 41.6% (*n* = 108) of the heifers presented BCS = 6 and RTS = 5, followed by 33.6% (*n* = 87) of the heifers categorized with BCS = 6 and RTS = 4 (Additional file 1: Table S1). Among different groups of BCS and RTS categories there were fewer open heifers (11%) among those classified with BCS = 6 and RTS = 5 (Additional file 1: Figure S4). The heifers were born to dams between 2 and 15 years of age with 53% born to dams two to four years old (Additional file 1: Figure S3).

**Table 2:** Percentages of pregnancy outcome by reproductive tract scoring and body condition scores

	*N*	Preg AI, %	Preg NS, %	Not preg, %
RTS				
3	20	30.0	50.0	20.0
4	104	46.2	35.6	18.3
5	135	42.2	45.2	12.6
BCS				
4	1	100.0	0.0	0.0
5	48	31.3	50.0	18.8
6	209	45.0	40.2	14.8
7	1	100.0	0.0	0.0

### Analysis of phenotypic parameters relative to heifer pregnancy outcome

Assessment of the full model indicated that weight at weaning, age at weaning, age at breeding, BCS, and RTS had dispensable contribution to the variability observed in the response variable, namely reproductive outcome (Additional file 1: Table S2). By comparison, the variables location and year presented significant (*P* = 0.0191 and *P* < 0.0001, respectively) contribution to the variance. The variable age of dam, although not significantly associated with pregnancy outcome, also contributed to a model that better fits the variance of the data [27].

**Fig. 1:**
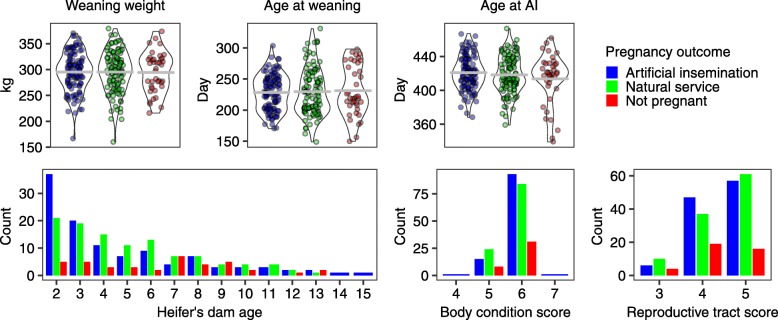
Distribution continuous and discrete variables evaluated in this study by pregnancy outcome in beef heifers

Figure 1 shows the distribution of the data for different parameters according to the reproductive outcome. Age or weight at weaning, age at breeding, dam age, BCS (Table 2), and RTS (Table 2) were not significantly associated with the response variable (*P* > 0.05), regardless of whether the logistic regression was carried with three (Preg AI, Preg NS, Not Preg; Table 3) or two (Preg, Not Preg; Table 4) reproductive outcomes. The results are strong indication that these parameters are not predictive of successful reproductive outcome in beef heifers that had been pre-selected as acceptable to enter the breeding season.

**Table 3:** Analysis of variance for the multinomial logistic regression of pregnancy outcome (PregAI, PregNS, NotPreg)

Variable	LR χ^2^	Df	Pr(>χ^2^)
Station	11.779	4	0.0191
AI year	37.266	10	< 0.0001
Age at AI	0.138	2	0.9335
Age at weaning	0.218	2	0.8967
Dam age	3.753	2	0.1532
BCS	3.405	6	0.7565
RTS	2.046	4	0.7273
Weaning weight	0.193	2	0.9079

*LR* Likelihood ratio, *Df* degrees of freedom, *Pr* probability

**Table 4:** Analysis of variance for the binomial logistic regression of pregnancy outcome (Preg, NotPreg)

Variable	LR χ^2^	Df	Pr(>χ^2^)
Station	7.3549	2	0.0253
AI year	23.7948	5	0.0002
Age at AI	0.0241	1	0.8766
Age at weaning	0.0366	1	0.8483
Dam age	1.4052	1	0.2359
BCS	1.3972	3	0.7062
RTS	0.8231	2	0.6626
Weaning weight	0.1682	1	0.6817

*LR* Likelihood ratio, *Df* degrees of freedom, *Pr* probability

It was noteworthy that no heifer younger than 368 d became pregnant by AI (Fig. 1). We further categorized our dataset based on heifers younger than 368 days of age, or heifers ≥368 days of age. We calculated an 87.5% probability of a heifer to become pregnant if she was 368 days of age or older at the beginning of the breeding season (odds = 7, 95% CI [2.7,17.9], *P* < 0.001). Contrariwise, there was only 12.5% chance of a heifer to become pregnant if she was younger than 368 d (odds = 0.14, 95% CI [0.06, 0.3], *P* < 0.001).

## Discussion

Optimizing the selection of beef replacement heifers is central for enhancing efficiency of the beef industry. Proper management practices serve to eliminate animals from this costly program and increase the likelihood of obtaining greater pregnancy rates early in the first breeding season. In this study, we analyzed key phenotypic and age profiles of Angus × Simmental heifers that were developed to become replacement heifers and were pre-selected for replacement potential prior to entering the breeding season. Our findings provide evidence-based insights on development and selection of beef heifers relative to their reproductive outcome.

Average weaning weight depicted in this study (294.7 ± 38.9 kg for heifers exposed to breeding) was greater than the average recorded weights of replacement beef heifers across the United States (241.3 kg [28]). This greater weight can be partly attributed to heifers being weaned, on average, 27 d (average of weaning age = 229.2 ± 34.3) older than the reported national average age at weaning (207 d [28]). The BCS, RTS, and ages of heifers at AI in this study are in agreement with recommended management practices for replacement heifer development [4, 29]. A greater than expected number of heifers were retained from two-year-old dams. However, the management practices of each station exclude artificial insemination of the mature cowherd, thus more animals were retained from first parity dams to increase genetic improvement at the experimental stations.

There was no association between age or weight at weaning and pregnancy outcomes. The results corroborate a metanalysis performed on beef heifers by Canellas and others [30]. These observations demonstrate a window of opportunity for the development of heifers of varying weaning weight to reach a target mature body weight greater than 53% that is likely influential on the reproductive performance [7].

Nearly all heifers included in this study were pre-selected prior to entering the breeding season according to general recommendations to increase pregnancy success [14]. Five heifers that entered the breeding season presenting RTS = 2 (4 became pregnant, 1 remained open) were removed during the data filtering to accomplish the goal of investigating a data set that adhered to best practices for improving pregnancy success in beef heifers [14]. Contrary to previous reports [9, 31], our analytical modeling did not detect significant association between RTS and reproductive outcome. Nevertheless, 46 and 42% of heifers presenting RTS 4 and 5, respectively, became pregnant to AI comparatively to the 30% classified with RTS 3. Although not statistically significant, there was a decline in open heifers at the end of the breeding season as the heifers presented greater RTS (Table 2).

Angus crossbred beef heifers presenting BCS ≥ 5 had greater pregnancy rates relative to heifers categorized with less fat percentage [4]. In our study, there was 14% difference on pregnancy rates to AI on heifers categorized with BCS = 6 relative to those classified with BCS = 5. Nonetheless, the final pregnancy rates were very similar on both groups (85% vs. 81%). Our results indicate that maintaining a nutritional program that allow heifers to reach ~ 22% of body fat (BCS = 6, [20]) at the beginning of the breeding season gave a numerical advantage on pregnancy success to AI (Table 2, Additional file 1: Figure S4). Beyond the quicker changes in the genetic background of the herd, the early conception to AI and early calving are determinant for greater longevity of the heifers in the breeding herd [32, 33].

Heifers younger than 368 days of age did not become pregnant by AI, and only 1 of these 6 young heifers became pregnant by natural service. It must be noted that the one heifer that became pregnant by natural service presented BCS = 5 and RTS = 3, while the others that remained open presented BCS = 6 and RTS ≥ 4. Although RTS indicated that these heifers had reached puberty, these data demonstrated that the age should be carefully assessed within the context of the production systems as a potential criterion for heifer culling.

## Conclusions

We report phenotypical parameters of beef heifers participant of replacement development programs in cow-calf production systems. On this group of pre-selected heifers, our analytical approach did not identify phenotypical or age-related parameters that are predictive of reproductive outcomes. However, it must be noted that developing heifers to BCS = 6 and RTS ≥ 4 might promote a numerical advantage of successful pregnancy to AI, supporting previous management suggestions. Careful risk assessment should be made when developing replacement heifers if they will not be older than 12 months of age by the start of the breeding season.

The data collected is restricted to *Bos taurus*, crossbred beef heifers (Angus × Simmental) on three research stations in the state of Alabama, thus it is difficult to evaluate how representative our results are of beef cow-calf systems of different biological types in different geographic areas in the USA. Nonetheless, our findings provide support for current management guidelines for the development of replacement beef heifers. More importantly, our limited ability to improve heifer pregnancy success from phenotypical parameters begs for the development of biotechnologies that will serve to reduce infertility in beef heifers.

## Additional files

Additional file 1: Supplementary information to the main document. (PDF 271 kb)

Additional file 2: Code used to analyze the data and obtain the results presented on this paper. (HTML 1819 kb)

## Abbreviations

adj WW: Adjusted weaning weight
AgeAI: Age at artificial insemination
AgeD: Dam age
AgeW: Age at weaning
AI: Artificial insemination
AIC: Akaike information criteria
BCS: Body condition score
CIDR: Controlled internal drug release
Df: Degrees of freedom
GnRH: Gonadotropin releasing hormone
kg: Kilogram
LR: Likelihood ratio
Not preg: Not pregnant
PGF: Prostaglandin F2α
Pr: Probability
Preg: Pregnant
Preg AI: Pregnant to artificial insemination
Preg NS: Pregnant to natural service
RTS: Reproductive tract score
USDA: United States Department of Agriculture
WW: Weaning weight
χ^2^: Chi-squared
