# Supplementary material for: Evaluation of age, weaning weight, body condition score, and reproductive tract score in pre-selected beef heifers relative to reproductive potential
Source: J Anim Sci Biotechnol. 2019 Feb 26;10:18. doi: 10.1186/s40104-019-0329-6 (PMC6390375; doi:10.1186/s40104-019-0329-6)
Supplement: Supplementary file 1 — Supplementary information to the main document. (PDF 271 kb) [file 40104_2019_329_MOESM1_ESM.pdf]

ADDITIONAL FILE 1

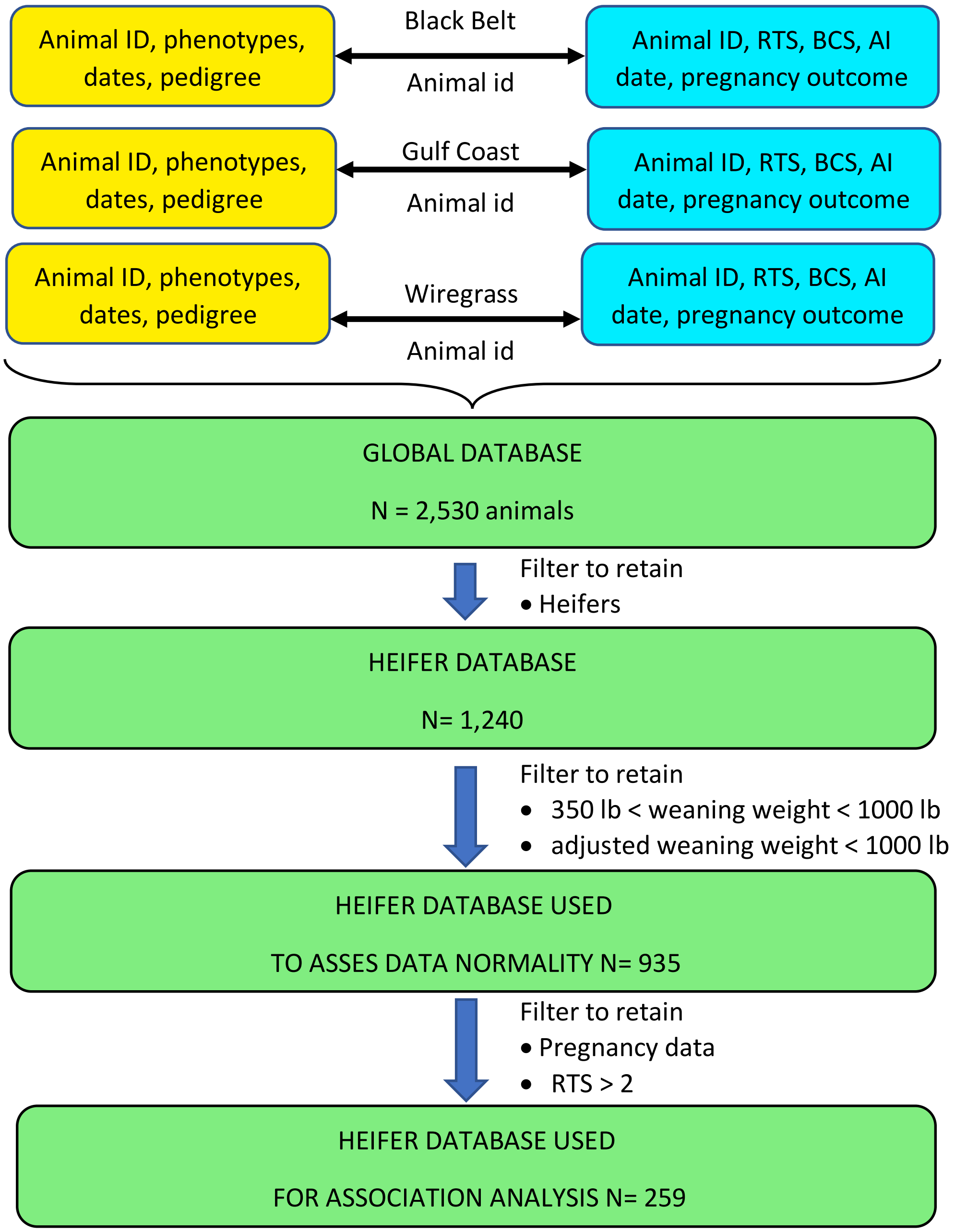

Figure S1. Schematics of the databases and filtering used in the current study.

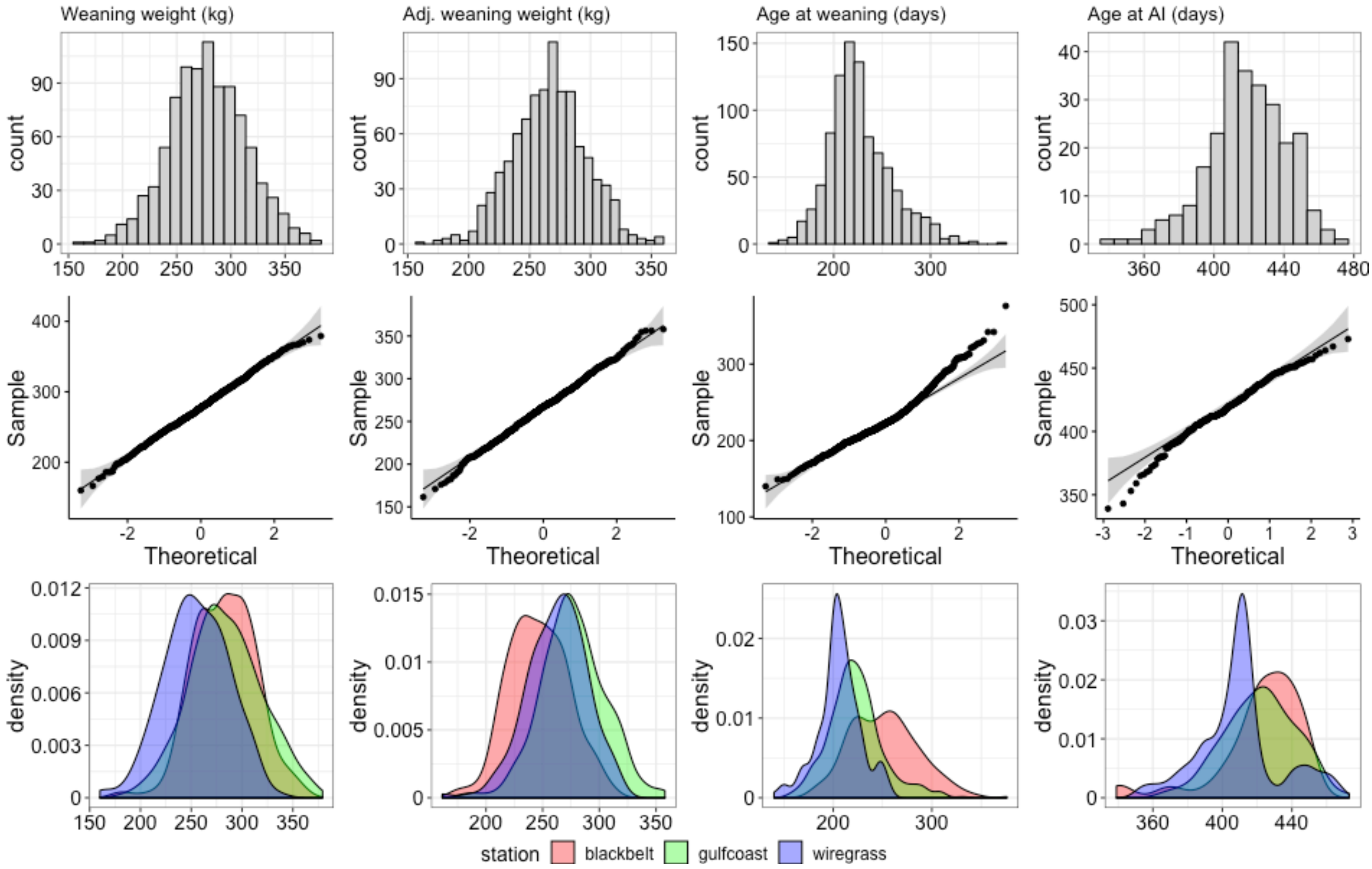

Figure S2. Distribution of the continuous variables investigated.

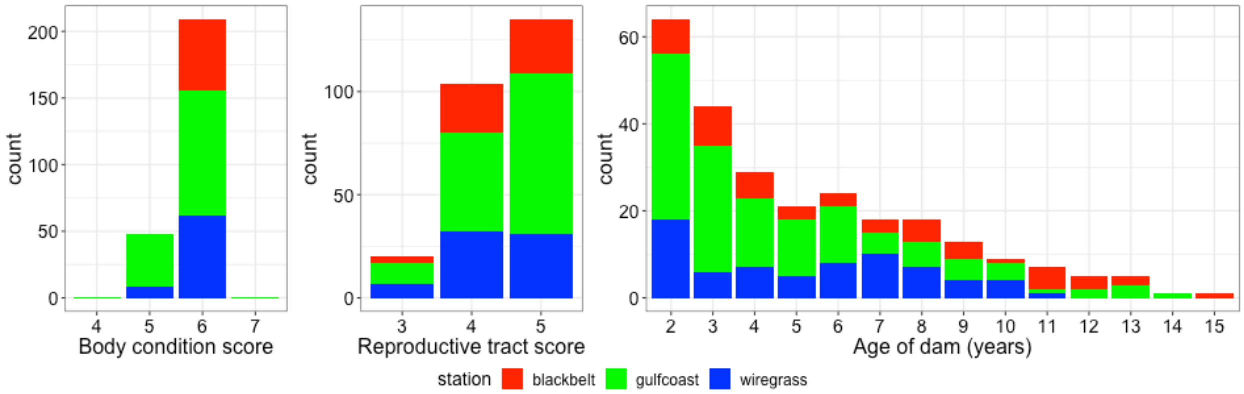

Figure S3. Distribution of the discrete variables investigated in this study.

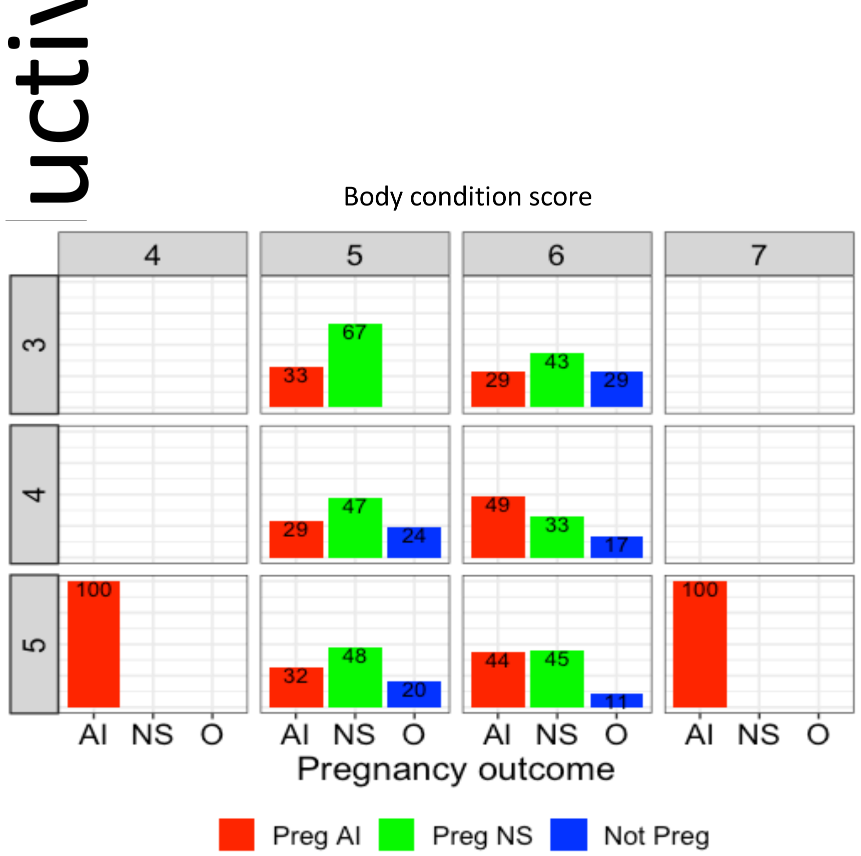

Figure S4. Percentages of reproductive outcome within different groups of beef heifers categorized by body condition score and reproductive tract score.

Table S1. Percentages of the heifers distributed on different categories of BCS and RTS.

		RTS		
		3	4	5
BCS	4	0.0	0.0	0.4
	5	2.3	6.6	9.7
	6	5.4	33.6	41.7
	7	0.0	0.0	0.4

Table S2. Stepwise assessment of the model utilized in our study by Akaike Information Criterion (AIC).

Step	Df	Deviance	RDf	RDev	AIC
			225	451.61	519.61
- BCS	6	3.41	231	455.01	511.01
- RTS	4	2.03	235	457.04	505.04
- Weaning weight	2	0.21	237	457.25	501.25
- Age at weaning	2	0.45	239	457.70	497.70
- Age at AI	2	0.25	241	457.95	493.95

Df: Degrees of freedom; RDf: Residual Degree of freedom, RDev: Residual Deviance
