# Supplementary material for: Evaluation of age, weaning weight, body condition score, and reproductive tract score in pre-selected beef heifers relative to reproductive potential
Source: J Anim Sci Biotechnol. 2019 Feb 26;10:18. doi: 10.1186/s40104-019-0329-6 (PMC6390375; doi:10.1186/s40104-019-0329-6)
Supplement: Supplementary file 2 — Code used to analyze the data and obtain the results presented on this paper. (HTML 1819 kb) [file 40104_2019_329_MOESM2_ESM.html]

Evaluation of age, weaning weight, body condition score, and reproductive tract score in pre-selected beef heifers relative to reproductive potential.


# Evaluation of age, weaning weight, body condition score, and reproductive tract score in pre-selected beef heifers relative to reproductive potential.

#### *Sarah E Dickinson, Michelle F Elmore, Lisa Kriese-Anderson, Joshua B Elmore, Bailey Walker, Paul W Dyce, Soren P Rodning, Fernando H Biase*

#### *September 2018*

Code created by Sarah Dickinson and Fernando Biase. Please, direct questions to Fernando Biase at *fbiase* at *auburn* dot *edu*.

Data availability: To obtain the data utilized on this study, please contact Dr. Michelle Elmore from the Alabama Beef Cattle Improvement Association at *elmormf* at *auburn* dot *edu*.

```
library(ggpubr)
library(ggplot2)
library(ggmosaic)
library(gridExtra)
library(ggrepel)
library(nnet)
library(pedigree)
library(kinship2)
library(gmodels)
library(afex)
library(car)
library(kableExtra)
library(dplyr)
library(MASS)
```

Load the data containig phenotypes, date of birth and other records and merge with the data on reproductive soundness and reproductive outcome.

```
#load and merge the data from Wiregrass
data_wiregrass<-read.csv("cattle-inventory_WG.csv",stringsAsFactors =FALSE)
data_wiregrass<-data_wiregrass[!(duplicated(data_wiregrass$Ear.Tag) | duplicated(data_wiregrass$Ear.Tag, fromLast = TRUE)), ]
data_preg_wiregrass<-read.delim("WREC_compiled.txt",stringsAsFactors =FALSE)
data_preg_wiregrass[duplicated(data_preg_wiregrass$ID),]
data_wiregrass<-merge(data_wiregrass,data_preg_wiregrass, by.x="Ear.Tag", by.y="ID",all.x=TRUE)
data_wiregrass$station<-"wiregrass"

#Load and merge the data for Gulf Coast
data_gulfcoast<-read.csv("cattle-inventory_GC.csv",stringsAsFactors =FALSE)
data_gulfcoast<-data_gulfcoast[!(duplicated(data_gulfcoast$Ear.Tag) | duplicated(data_gulfcoast$Ear.Tag, fromLast = TRUE)), ]
data_preg_gulfcoast<-read.delim("GCREC_compiled.txt",stringsAsFactors =FALSE)
data_preg_gulfcoast[duplicated(data_preg_gulfcoast$ID),]
data_gulfcoast<-merge(data_gulfcoast,data_preg_gulfcoast, by.x="Ear.Tag", by.y="ID",all.x=TRUE)
data_gulfcoast$station<-"gulfcoast"

#Load and merge the data for BlackBelt
data_blackbelt<-read.csv("cattle-inventory_BB.csv",stringsAsFactors =FALSE)
data_blackbelt<-data_blackbelt[!(duplicated(data_blackbelt$Ear.Tag) | duplicated(data_blackbelt$Ear.Tag, fromLast = TRUE)), ]
data_preg_blackbelt<-read.delim("BBREC_compiled.txt",stringsAsFactors =FALSE)
data_preg_blackbelt[duplicated(data_preg_blackbelt$ID),]
data_blackbelt<-merge(data_blackbelt,data_preg_blackbelt, by.x="Ear.Tag", by.y="ID",all.x=TRUE)
data_blackbelt$station<-"blackbelt"

#merge the three stations into one sheet
data_stations<-rbind(data_wiregrass,data_gulfcoast,data_blackbelt)
```

Number of observations on the unfiltered dataset.

```
dim(data_stations)[1]
```

```
## [1] 2530
```

Reformat the dates, and calculate the variables: Age at weaning (days), Age at Artificial Insemination (days), Age of the Dam (years). Also convert the weight data from pounds (lb) to kilograms (kg).

```
#calculate days to weaning/AI and convert lb to kg
data_stations$Birth.Date.2 <- gsub('-', '/', data_stations$Birth.Date)
data_stations$Weaning.Date.2 <- gsub('-', '/', data_stations$Weaning.Date)
data_stations$Dam.Birth.Date.2 <- gsub('-', '/', data_stations$Dam.Birth.Date)
data_stations$AI_date.2 <- gsub('-', '/', data_stations$AI_DATE)
data_stations$AI_date.2 <- gsub('/201', '/1', data_stations$AI_date.2)
data_stations$Birth.Date.3<-as.Date(data_stations$Birth.Date.2,format = "%m/%d/%Y")
data_stations$Weaning.Date.3<-as.Date(data_stations$Weaning.Date.2,format = "%m/%d/%Y")
data_stations$Dam.Birth.Date.3<-as.Date(data_stations$Dam.Birth.Date.2,format = "%m/%d/%Y")
data_stations$Date.AI.3<-as.Date(data_stations$AI_date.2,format = "%m/%d/%Y")
data_stations$Year<-substring(data_stations$Birth.Date.3, 1,4)
data_stations$Dam.Birth.Year<-substring(data_stations$Dam.Birth.Date.3, 1,4)
data_stations$Dam.Birth.Year.2 <- gsub('0097', '1997', data_stations$Dam.Birth.Year) 
data_stations$Dam.Birth.Year.2 <- gsub('0098', '1998', data_stations$Dam.Birth.Year.2) 
data_stations$Dam.Birth.Year.2 <- gsub('0099', '1999', data_stations$Dam.Birth.Year.2)
data_stations$Dam.Birth.Year.2 <- gsub('0000', '2000', data_stations$Dam.Birth.Year.2) 
data_stations$Dam.Birth.Year.2 <- gsub('0001', '2001', data_stations$Dam.Birth.Year.2) 
data_stations$Dam.Birth.Year.2 <- gsub('0002', '2002', data_stations$Dam.Birth.Year.2) 
data_stations$Dam.Birth.Year.2 <- gsub('0003', '2003', data_stations$Dam.Birth.Year.2) 
data_stations$Dam.Birth.Year.2 <- gsub('0004', '2004', data_stations$Dam.Birth.Year.2) 
data_stations$Dam.Birth.Year.2 <- gsub('0005', '2005', data_stations$Dam.Birth.Year.2) 
data_stations$Dam.Birth.Year.2 <- gsub('0006', '2006', data_stations$Dam.Birth.Year.2) 
data_stations$Dam.Birth.Year.2 <- gsub('0007', '2007', data_stations$Dam.Birth.Year.2) 
data_stations$Dam.Birth.Year.2 <- gsub('0008', '2008', data_stations$Dam.Birth.Year.2) 
data_stations$Dam.Birth.Year.2 <- gsub('0009', '2009', data_stations$Dam.Birth.Year.2) 
data_stations$Dam.Birth.Year.2 <- gsub('0010', '2010', data_stations$Dam.Birth.Year.2) 
data_stations$Dam.Birth.Year.2 <- gsub('0011', '2011', data_stations$Dam.Birth.Year.2) 
data_stations$Dam.Birth.Year.2 <- gsub('0012', '2012', data_stations$Dam.Birth.Year.2) 
data_stations$Dam.Birth.Year.2 <- gsub('0013', '2013', data_stations$Dam.Birth.Year.2) 
data_stations$Dam.Birth.Year.2 <- gsub('0014', '2014', data_stations$Dam.Birth.Year.2) 
data_stations$Year.2 <- gsub('001', '201', data_stations$Year) 
data_stations$AI_Year<-substring(data_stations$Date.AI.3, 3,4)
data_stations$Birth_to_Weaning<-difftime(data_stations$Weaning.Date.3, data_stations$Birth.Date.3, units="days")
data_stations$Birth_to_AI<-difftime(data_stations$Date.AI.3, data_stations$Birth.Date.3, units="days")
data_stations$Weaning_to_AI<-difftime(data_stations$Date.AI.3, data_stations$Weaning.Date.3, units="days")
data_stations$Dam.Age<- as.integer(data_stations$Year.2) - as.integer(data_stations$Dam.Birth.Year.2)
data_stations$Birth_to_Weaning <- as.integer(gsub(' days', '', data_stations$Birth_to_Weaning))
data_stations$Birth_to_AI <- as.integer(gsub(' days', '', data_stations$Birth_to_AI))
data_stations$Weaning_to_AI <- as.integer(gsub(' days', '', data_stations$Weaning_to_AI))
data_stations$Adj.Birth.Weight.Kg<-data_stations$Adj.Birth.Weight*0.453592
data_stations$Adj.Weaning.Weight.Kg<-data_stations$Adj.Weaning.Weight*0.453592
data_stations$Birth.Weight.Kg<-data_stations$Birth.Weight*0.453592
data_stations$Weaning.Weight.Kg<-data_stations$Weaning.Weight*0.453592
```

Filter the dataset to retain records on heifers only, and output the number of records retained.

```
data_stations<-data_stations[!is.na(data_stations$station),]
data_stations<-data_stations[data_stations$Sex=="Heifer",]
```

Number of observations on dataset filtered to retain data on heifers.

```
dim(data_stations)[1]
```

```
## [1] 1240
```

Filter the dataset to retain heifers with weaning weight between 350 and 1000 lb.

```
data_stations_a<-data_stations
data_stations_a<-data_stations_a[!is.na(data_stations_a$Weaning.Weight),]
data_stations_a<-data_stations_a[!is.na(data_stations_a$station),]
data_stations_a<-data_stations_a[data_stations_a$Weaning.Weight > 350 ,]
data_stations_a<-data_stations_a[data_stations_a$Weaning.Weight < 1000 ,]
data_stations_a<-data_stations_a[data_stations_a$Adj.Weaning.Weight < 1000 ,]
```

Number of observations on dataset filtered.

```
dim(data_stations_a)[1]
```

```
## [1] 935
```

Filter the dataset to retain heifers with Reproductive tract score greater than 2 and with pregnancy data.

```
data_stations_b<-data_stations_a
data_stations_b<-data_stations_b[data_stations_b$RTS >2 ,]
data_stations_b<-data_stations_b[!is.na(data_stations_b$PREG_OUTCOME),]
```

Number of observations on dataset filtered.

```
dim(data_stations_b)[1]
```

```
## [1] 259
```

Obtain the resulst used to create table 1.

```
weaning_weight_all_heifers<-aggregate(Weaning.Weight.Kg ~ Weaning.Weight.Kg , data_stations_a, function(x) c(mean = round(mean(x),1), sd = round(sd(x),1),  round(ci(x, confidence=0.95)[2],1), round(ci(x, confidence=0.95)[3],1) ))
weaning_weight_AI_heifers<-aggregate(Weaning.Weight.Kg ~ Weaning.Weight.Kg , data_stations_b, function(x) c(mean = round(mean(x),1), sd = round(sd(x),1),  round(ci(x, confidence=0.95)[2],1), round(ci(x, confidence=0.95)[3],1) ))
adj_weaning_weight_all_heifers<-aggregate(Adj.Weaning.Weight.Kg ~ Adj.Weaning.Weight.Kg , data_stations_a, function(x) c(mean = round(mean(x),1), sd = round(sd(x),1),  round(ci(x, confidence=0.95)[2],1), round(ci(x, confidence=0.95)[3],1) ))
adj_weaning_weight_AI_heifers<-aggregate(Adj.Weaning.Weight.Kg ~ Adj.Weaning.Weight.Kg , data_stations_b, function(x) c(mean = round(mean(x),1), sd = round(sd(x),1),  round(ci(x, confidence=0.95)[2],1), round(ci(x, confidence=0.95)[3],1)))
age_at_weaning_weight_all_heifers<-aggregate(Birth_to_Weaning ~ Birth_to_Weaning , data_stations_a, function(x) c(mean = round(mean(x),1), sd = round(sd(x),1),  round(ci(x, confidence=0.95)[2],1), round(ci(x, confidence=0.95)[3],1) ))
age_at_weaning_weight_AI_heifers<-aggregate(Birth_to_Weaning ~ Birth_to_Weaning , data_stations_b, function(x) c(mean = round(mean(x),1), sd = round(sd(x),1),  round(ci(x, confidence=0.95)[2],1), round(ci(x, confidence=0.95)[3],1) ))
age_at_AI_heifers<-aggregate(Birth_to_AI ~ Birth_to_AI , data_stations_b, function(x) c(mean = round(mean(x),1), sd = round(sd(x),1),  round(ci(x, confidence=0.95)[2],1), round(ci(x, confidence=0.95)[3],1) ))
```

Table 1. Descriptive statistics of continuous variables from beef heifers.

```
table1<-cbind(data.frame(Variable=c(rep("Weaning weight (kg)",2), rep("Adj weaning weight (kg)",2), rep("Age at weaning (days)",2), "Age at AI (days)"), 
                   Dataset=c(rep(c("All heifers","Pregnancy heifers"),3), "Pregnancy heifers"),
                   No.Records=c(rep(c(935,259),3),259))
              ,
                   rbind(weaning_weight_all_heifers[,1],weaning_weight_AI_heifers[,1],adj_weaning_weight_all_heifers[,1],adj_weaning_weight_AI_heifers[,1],age_at_weaning_weight_all_heifers[,1],age_at_weaning_weight_AI_heifers[,1],age_at_AI_heifers[,1]))

kable(table1) %>%
  kable_styling(full_width = F) %>%
  collapse_rows(columns = 1, valign = "top")
```

| Variable | Dataset | No.Records | mean | sd | CI lower | CI upper |
| --- | --- | --- | --- | --- | --- | --- |
| Weaning weight (kg) | All heifers | 935 | 278.0 | 35.5 | 275.8 | 280.3 |
|  | Pregnancy heifers | 259 | 294.7 | 38.9 | 289.9 | 299.4 |
| Adj weaning weight (kg) | All heifers | 935 | 266.2 | 30.4 | 264.2 | 268.1 |
|  | Pregnancy heifers | 259 | 278.7 | 26.6 | 275.5 | 282.0 |
| Age at weaning (days) | All heifers | 935 | 227.2 | 32.6 | 225.1 | 229.3 |
|  | Pregnancy heifers | 259 | 229.2 | 34.3 | 225.0 | 233.4 |
| Age at AI (days) | Pregnancy heifers | 259 | 418.7 | 22.6 | 416.0 | 421.5 |

Statistical assessment of normality of the continuous variables.

```
#Weaning weight
shapiro.test(data_stations_a$Weaning.Weight)
```

```
## 
##  Shapiro-Wilk normality test
## 
## data:  data_stations_a$Weaning.Weight
## W = 0.99869, p-value = 0.7352
```

```
#Age at weaning (days)
shapiro.test(data_stations_a$Birth_to_Weaning)
```

```
## 
##  Shapiro-Wilk normality test
## 
## data:  data_stations_a$Birth_to_Weaning
## W = 0.96863, p-value = 2.598e-13
```

```
#Age at artificial inseination (days)
shapiro.test(data_stations_b$Birth_to_AI)
```

```
## 
##  Shapiro-Wilk normality test
## 
## data:  data_stations_b$Birth_to_AI
## W = 0.98305, p-value = 0.003569
```

Create the chart for visual inspection of the continuous variables.

```
fmt_dcimals <- function(decimals=0){
  # return a function responpsible for formatting the 
  # axis labels with a given number of decimals 
  function(x) as.character(round(x,decimals))
}

plot1<-ggplot(data=data_stations_a,aes(x=Weaning.Weight.Kg)) +
  geom_histogram(colour="black", fill="lightgray",bins=23)+
  labs(title="Weaning weight (kg)")+
  theme_bw()+
  theme(axis.text = element_text(size = 12, color="black"),
        axis.title = element_text(size = 14, color="black"),
        axis.title.x = element_blank())

plot2<-ggqqplot(data_stations_a, x="Weaning.Weight.Kg")+
  font("xlab", size =16, color="black")+
  font("ylab", size =16, color="black")

plot3<-ggplot(data=data_stations_a,aes(x=Weaning.Weight.Kg, fill=station)) +
  geom_density(alpha=.4)+
  scale_y_continuous(labels = fmt_dcimals(4))+
  scale_fill_manual(values=c("red", "green", "blue"))+
  theme_bw()+
  theme(axis.text = element_text(size = 12, color="black"),
        axis.title = element_text(size = 14, color="black"),
        legend.position="none",
        axis.title.x = element_blank())

plot4<-ggplot(data=data_stations_a,aes(x=Adj.Weaning.Weight.Kg)) +
  geom_histogram(colour="black", fill="lightgray", bins=28)+
  labs(title="Adj. weaning weight (kg)")+
  theme_bw()+
  theme(axis.text = element_text(size = 12, color="black"),
        axis.title = element_text(size = 14, color="black"),
        axis.title.x = element_blank())

plot5<-ggqqplot(data_stations_a, x="Adj.Weaning.Weight.Kg")+
  font("xlab", size =16, color="black")+
  font("ylab", size =16, color="black")

plot6<-ggplot(data=data_stations_a,aes(x=Adj.Weaning.Weight.Kg, fill=station)) +
  geom_density(alpha=.4)+
  scale_y_continuous(labels = fmt_dcimals(4))+
  scale_fill_manual(values=c("red", "green", "blue"))+
  theme_bw()+
  theme(axis.text = element_text(size = 12, color="black"),
        axis.title = element_text(size = 14, color="black"),
        legend.position="none",
        axis.title.x = element_blank())

plot7<-ggplot(data=data_stations_a,aes(x=Birth_to_Weaning)) +
  geom_histogram(colour="black", fill="lightgray", bins=25)+
  labs(title="Age at weaning (days)")+
  theme_bw()+
  theme(axis.text = element_text(size = 12, color="black"),
        axis.title = element_text(size = 14, color="black"),
        axis.title.x = element_blank())

plot8<-ggqqplot(data_stations_a, x="Birth_to_Weaning")+
  font("xlab", size =16, color="black")+
  font("ylab", size =16, color="black")

plot9<-ggplot(data=data_stations_a,aes(x=Birth_to_Weaning, fill=station)) +
  geom_density(alpha=.4)+
  scale_y_continuous(labels = fmt_dcimals(4))+
  scale_fill_manual(values=c("red", "green", "blue"))+
  theme_bw()+
  theme(axis.text = element_text(size = 12, color="black"),
        axis.title = element_text(size = 14, color="black"),
        legend.position="none",
        axis.title.x = element_blank())

plot10<-ggplot(data=data_stations_b,aes(x=Birth_to_AI)) +
  geom_histogram(colour="black", fill="lightgray", bins=18)+
  labs(title="Age at AI (days)")+
  theme_bw()+
  theme(axis.text = element_text(size = 12, color="black"),
        axis.title = element_text(size = 14, color="black"),
        axis.title.x = element_blank())

plot11<-ggqqplot(data_stations_b, x="Birth_to_AI")+
  font("xlab", size =16, color="black")+
  font("ylab", size =16, color="black")

plot12<-ggplot(data=data_stations_b,aes(x=Birth_to_AI, fill=station)) +
  geom_density(alpha=.4)+
  scale_y_continuous(labels = fmt_dcimals(4))+
  scale_fill_manual(values=c("red", "green", "blue"))+
  theme_bw()+
  theme(axis.text = element_text(size = 12, color="black"),
        axis.title = element_text(size = 14, color="black"),
        legend.position="bottom",
        legend.text = element_text(size = 12, color="black"),
        legend.title = element_text(size = 12, color="black"))

get_legend<-function(myggplot){
  tmp <- ggplot_gtable(ggplot_build(myggplot))
  leg <- which(sapply(tmp$grobs, function(x) x$name) == "guide-box")
  legend <- tmp$grobs[[leg]]
  return(legend)
}
legend <- get_legend(plot12)

plot12<-ggplot(data=data_stations_b,aes(x=Birth_to_AI, fill=station)) +
  geom_density(alpha=.4)+
  scale_y_continuous(labels = fmt_dcimals(4))+
  scale_fill_manual(values=c("red", "green", "blue"))+
  theme_bw()+
  theme(legend.title=element_text(size=12), legend.text=element_text(size=12), axis.text = element_text(size = 12, color="black"),
        axis.title = element_text(size = 12, color="black"),
        legend.position="none",
        axis.title.x = element_blank())
```

Figure S2. Distribution of the continuous variables investigated.

```
grid.arrange(plot1, plot4, plot7, plot10, plot2, plot5, plot8, plot11, plot3, plot6, plot9, plot12, legend, ncol=4, nrow = 4, 
             layout_matrix = rbind(c(1,2,3,4), c(5,6,7,8), c(9, 10, 11, 12), c(13, 13, 13, 13)),
             widths = c(2.7, 2.7, 2.7, 2.7), heights = c(2.5, 2.5, 2.3,0.2))
```

Create the chart for visual inspection of the discrete variables.

```
Plot13<-ggplot(data_stations_b, aes(x=factor(BCS), fill=station))+
  geom_bar(stat="count")+
  scale_x_discrete("Body condition score")+
  scale_fill_manual(values=c("red", "green", "blue"))+
  theme_bw()+
  theme(axis.text = element_text(size = 12, color="black"),
        axis.title = element_text(size = 12, color="black") ,
        legend.position="none")


Plot14<-ggplot(data_stations_b, aes(x=factor(RTS), fill=station))+
  geom_bar(stat="count")+
  scale_x_discrete("Reproductive tract score")+
  scale_fill_manual(values=c("red", "green", "blue"))+
  theme_bw()+
  theme(axis.text = element_text(size = 12, color="black"),
        axis.title = element_text(size = 12, color="black"),
        legend.position="none")

Plot15<-ggplot(data_stations_b, aes(x=factor(Dam.Age), fill=station))+
  geom_bar(stat="count")+
  scale_x_discrete("Age of dam (years)")+
  scale_fill_manual(values=c("red", "green", "blue"))+
  theme_bw()+
  theme(axis.text = element_text(size = 12, color="black"),
        axis.title = element_text(size = 12, color="black") ,
        legend.position="bottom",
        legend.text = element_text(size = 12, color="black"),
        legend.title = element_text(size = 12, color="black"))

get_legend<-function(myggplot){
  tmp <- ggplot_gtable(ggplot_build(myggplot))
  leg <- which(sapply(tmp$grobs, function(x) x$name) == "guide-box")
  legend <- tmp$grobs[[leg]]
  return(legend)
}
legend <- get_legend(Plot15)


Plot15<-ggplot(data_stations_b, aes(x=factor(Dam.Age), fill=station))+
  geom_bar(stat="count")+
  scale_x_discrete("Age of dam (years)")+
  scale_fill_manual(values=c("red", "green", "blue"))+
  theme_bw()+
  theme(axis.text = element_text(size = 12, color="black"),
        axis.title = element_text(size = 12, color="black") ,
        legend.position="none")
```

Figure S3. Distribution of the discrete variables investigated in this study.

```
grid.arrange(Plot13, Plot14, Plot15, legend, ncol=3, nrow = 2, 
             layout_matrix = rbind(c(1,2,3), c(4,4,4)),
             widths = c(1.8, 1.8, 4), heights = c(2.5,0.2))
```

Table S1. Percentages of the heifers distributed on different categories of BCS and RTS.

```
kable(
as.data.frame.matrix(round(prop.table(table(data_stations_b$BCS,data_stations_b$RTS))*100,1))
) %>%
kable_styling(full_width = F) %>%
  add_header_above(c(" ", "RTS" = 3))%>%
   column_spec(1, bold = T, color="black")
```

|  | RTS | | |
| --- | --- | --- | --- |
|  | 3 | 4 | 5 |
| 4 | 0.0 | 0.0 | 0.4 |
| 5 | 2.3 | 6.6 | 9.7 |
| 6 | 5.4 | 33.6 | 41.7 |
| 7 | 0.0 | 0.0 | 0.4 |

Table 2. Percentages of pregnancy outcome by reproductive tract scoring and body condition scores.

```
RTS_BCS_matrix<-rbind(
  round(prop.table(table(data_stations_b$RTS,data_stations_b$PREG_OUTCOME),margin=1)*100,1),
  round(prop.table(table(data_stations_b$BCS,data_stations_b$PREG_OUTCOME),margin=1)*100,1)
     )
RTS_BCS_matrix<-cbind(RTS_BCS_matrix,c(20, 204, 135, 1, 48, 209, 1))
RTS_BCS_matrix<-RTS_BCS_matrix[,c(4,1:3)]
colnames(RTS_BCS_matrix)<-c("N", "Preg AI(%)", "Preg NS(%)", "Not Preg(%)")

kable(RTS_BCS_matrix) %>%
kable_styling(full_width = F) %>%
  group_rows("RTS",1,3) %>%
  group_rows("BCS",4,7)
```

|  | N | Preg AI(%) | Preg NS(%) | Not Preg(%) |
| --- | --- | --- | --- | --- |
| **RTS** | | | | |
| 3 | 20 | 30.0 | 50.0 | 20.0 |
| 4 | 204 | 46.2 | 35.6 | 18.3 |
| 5 | 135 | 42.2 | 45.2 | 12.6 |
| **BCS** | | | | |
| 4 | 1 | 100.0 | 0.0 | 0.0 |
| 5 | 48 | 31.2 | 50.0 | 18.8 |
| 6 | 209 | 45.0 | 40.2 | 14.8 |
| 7 | 1 | 100.0 | 0.0 | 0.0 |

Table 3 Analysis of variance (type III) for the multinomial logistic regression of pregnancy outcome (Preg AI, Preg NS, Not Preg) on phenotypic parameters.

```
data_stations_b$station<-factor(data_stations_b$station)
data_stations_b$AI_Year<-factor(data_stations_b$AI_Year)
data_stations_b$PREG_OUTCOME<-factor(data_stations_b$PREG_OUTCOME, levels=c("AI", "NS", "O"))
data_stations_b$BCS<-factor(data_stations_b$BCS)
data_stations_b$RTS<-factor(data_stations_b$RTS)
afex::set_sum_contrasts()

data_stations_b$PREG_OUTCOME<-relevel(as.factor(data_stations_b$PREG_OUTCOME), ref="O")
test <- multinom(PREG_OUTCOME ~    station + AI_Year + Birth_to_AI + Birth_to_Weaning  +   Dam.Age +BCS + RTS +  Weaning.Weight.Kg , data = data_stations_b,  Hess = TRUE)
```

```
Anova(test,type=c("III"))
```

```
## Analysis of Deviance Table (Type III tests)
## 
## Response: PREG_OUTCOME
##                   LR Chisq Df Pr(>Chisq)    
## station             11.779  4    0.01908 *  
## AI_Year             37.266 10  5.089e-05 ***
## Birth_to_AI          0.138  2    0.93347    
## Birth_to_Weaning     0.218  2    0.89673    
## Dam.Age              3.753  2    0.15315    
## BCS                  3.405  6    0.75653    
## RTS                  2.046  4    0.72734    
## Weaning.Weight.Kg    0.193  2    0.90794    
## ---
## Signif. codes:  0 '***' 0.001 '**' 0.01 '*' 0.05 '.' 0.1 ' ' 1
```

Table S2. Stepwise assessment of the model utilized in our study by Akaike Information Criterion (AIC).

```
step <- stepAIC(test, direction="both")
```

```
step$anova
```

```
## Stepwise Model Path 
## Analysis of Deviance Table
## 
## Initial Model:
## PREG_OUTCOME ~ station + AI_Year + Birth_to_AI + Birth_to_Weaning + 
##     Dam.Age + BCS + RTS + Weaning.Weight.Kg
## 
## Final Model:
## PREG_OUTCOME ~ station + AI_Year + Dam.Age
## 
## 
##                  Step Df  Deviance Resid. Df Resid. Dev      AIC
## 1                                        225   451.6070 519.6070
## 2               - BCS  6 3.4052238       231   455.0122 511.0122
## 3               - RTS  4 2.0271457       235   457.0393 505.0393
## 4 - Weaning.Weight.Kg  2 0.2115650       237   457.2509 501.2509
## 5  - Birth_to_Weaning  2 0.4486729       239   457.6996 497.6996
## 6       - Birth_to_AI  2 0.2502638       241   457.9498 493.9498
```

Table 4. Analysis of variance (type III) for the binomial logistic regression of pregnancy outcome (Preg, Not Preg) on phenotypic parameters.

```
data_stations_b$PREG_OUTCOME_coded<-ifelse(data_stations_b$PREG_OUTCOME == "AI", "Preg", ifelse(data_stations_b$PREG_OUTCOME == "NS", "Preg", "O"))
data_stations_b$PREG_OUTCOME_coded<-factor(data_stations_b$PREG_OUTCOME_coded)
test <- multinom(PREG_OUTCOME_coded ~   station + AI_Year + Birth_to_AI + Birth_to_Weaning  +   Dam.Age +BCS + RTS +  Weaning.Weight.Kg, data = data_stations_b,  Hess = TRUE)
```

```
Anova(test,type=c("III"),test.statistic="LR")
```

```
## Analysis of Deviance Table (Type III tests)
## 
## Response: PREG_OUTCOME_coded
##                   LR Chisq Df Pr(>Chisq)    
## station             7.3549  2  0.0252876 *  
## AI_Year            23.7948  5  0.0002377 ***
## Birth_to_AI         0.0241  1  0.8766165    
## Birth_to_Weaning    0.0366  1  0.8483036    
## Dam.Age             1.4052  1  0.2358513    
## BCS                 1.3972  3  0.7061861    
## RTS                 0.8231  2  0.6626367    
## Weaning.Weight.Kg   0.1682  1  0.6817004    
## ---
## Signif. codes:  0 '***' 0.001 '**' 0.01 '*' 0.05 '.' 0.1 ' ' 1
```

Figure 1. Distribution continuous and discrete variables evaluated in this study by pregnancy outcome in beef heifers.

```
data_stations_b<-data_stations_a
data_stations_b<-data_stations_b[data_stations_b$RTS >2 ,]
data_stations_b<-data_stations_b[!is.na(data_stations_b$PREG_OUTCOME),]

font_size<-9
symbol_size<-1

plot16<-ggplot(data=data_stations_b,aes(y=Weaning.Weight.Kg, x=PREG_OUTCOME))+
  #geom_boxplot()+
  geom_jitter(width = 0.2, shape=21, size=symbol_size, alpha=0.5, aes(fill=PREG_OUTCOME))+
  geom_violin(trim=TRUE, fill=NA,size=0.2)+
  scale_fill_manual(name=NULL,values=c("blue", "green", "red" ))+
  stat_summary(fun.y = mean, fun.ymin = mean, fun.ymax = mean, colour = "gray", size = 0.3,  geom = "crossbar", alpha=0.5)+
  scale_y_continuous(name="kg")+
  scale_x_discrete( labels = c('Preg AI','Preg NS','Not preg'))+
  theme_bw()+
  ggtitle("Weaning weight")+
  theme(axis.text = element_text(size = font_size, color="black"),
        axis.text.x = element_blank(),
        axis.title = element_text(size = font_size, color="black"),
        axis.title.x = element_blank(),
        plot.title = element_text(lineheight=.8, size = font_size, color="black",hjust = 0.5),
        panel.grid= element_blank(),
        panel.background = element_blank(),
        panel.grid.minor = element_blank(), 
        panel.grid.major = element_blank(),
        plot.background = element_blank(),
        legend.position="none",
        axis.ticks.x=element_blank())

plot17<-ggplot(data=data_stations_b,aes(y=Birth_to_Weaning, x=PREG_OUTCOME))+
  #geom_boxplot()+
  geom_jitter(width = 0.2, shape=21, size=symbol_size,alpha=0.5, aes(fill=PREG_OUTCOME))+
  geom_violin(trim=TRUE, fill=NA,size=0.2)+
  scale_fill_manual(name=NULL,values=c("blue", "green", "red" ))+
  stat_summary(fun.y = mean, fun.ymin = mean, fun.ymax = mean, colour = "gray", size = 0.3,  geom = "crossbar", alpha=0.5)+
  scale_y_continuous(name="days")+
  scale_x_discrete( labels = c('Preg AI','Preg NS','Not preg'))+
  theme_bw()+
  ggtitle("Age at weaning")+
  theme(axis.text = element_text(size = font_size, color="black"),
        axis.text.x = element_blank(),
        axis.title = element_text(size = font_size, color="black"),
        axis.title.x = element_blank(),
        plot.title = element_text(lineheight=.8, size = font_size, color="black",hjust = 0.5),
        panel.grid= element_blank(),
        panel.background = element_blank(),
        panel.grid.minor = element_blank(), 
        panel.grid.major = element_blank(),
        plot.background = element_blank(),
        legend.position="none",
        axis.ticks.x=element_blank())

plot18<-ggplot(data=data_stations_b,aes(y=Birth_to_AI, x=PREG_OUTCOME))+
  #geom_boxplot()+
  geom_jitter(width = 0.2, shape=21, size=symbol_size,alpha=0.5, aes(fill=PREG_OUTCOME))+
  geom_violin(trim=TRUE, fill=NA,size=0.2)+
  scale_fill_manual(name=NULL,values=c("blue", "green", "red" ))+
  stat_summary(fun.y = mean, fun.ymin = mean, fun.ymax = mean, colour = "gray", size = 0.3,  geom = "crossbar", alpha=0.5)+
  scale_y_continuous(name="days")+
  scale_x_discrete( labels = c('Preg AI','Preg NS','Not preg'))+
  theme_bw()+
  ggtitle("Age at AI")+
  theme(axis.text = element_text(size = font_size, color="black"),
        axis.text.x = element_blank(),
        axis.title = element_text(size = font_size, color="black"),
        axis.title.x = element_blank(),
        plot.title = element_text(lineheight=.8, size = font_size, color="black",hjust = 0.5),
        panel.grid= element_blank(),
        panel.background = element_blank(),
        panel.grid.minor = element_blank(), 
        panel.grid.major = element_blank(),
        plot.background = element_blank(),
        legend.position="none",
        axis.ticks.x=element_blank())

plot19<-ggplot(data_stations_b, aes(x=factor(Dam.Age), fill=PREG_OUTCOME))+
  geom_bar(stat="count",position="dodge2")+
  scale_x_discrete("Heifer's dam age")+
  scale_fill_manual(values=c("blue", "green", "red"))+
  theme_bw()+
  theme(panel.grid= element_blank(),
        axis.text = element_text(size = font_size, color="black"),
        axis.title = element_text(size = font_size, color="black"),
        legend.position="none")

plot20<-ggplot(data_stations_b, aes(x=BCS, fill=PREG_OUTCOME))+
  geom_bar(stat="count",position="dodge2")+
  scale_x_discrete("Body condition score")+
  scale_fill_manual(values=c("blue", "green", "red"))+
  theme_bw()+
  theme(panel.grid= element_blank(),
        axis.text = element_text(size = font_size, color="black"),
        axis.title = element_text(size = font_size, color="black"),
        legend.position="none")

plot21<-ggplot(data_stations_b, aes(x=RTS, fill=PREG_OUTCOME))+
  geom_bar(stat="count",position="dodge2")+
  scale_x_discrete("Reproductive tract score")+
  scale_fill_manual("Pregnancy outcome",labels=c("Artificial insemination", "Natural service", "Not pregnant"), values=c("blue", "green", "red"))+
  theme_bw()+
  theme(axis.text = element_text(size = font_size, color="black"),
        axis.title = element_text(size = font_size, color="black"),
        legend.position="right",
        legend.text = element_text(size = 9, color="black"),
        legend.title = element_text(size = 9, color="black"),
        legend.key.size = unit(3,"mm"))

get_legend<-function(myggplot){
  tmp <- ggplot_gtable(ggplot_build(myggplot))
  leg <- which(sapply(tmp$grobs, function(x) x$name) == "guide-box")
  legend <- tmp$grobs[[leg]]
  return(legend)
}
legend <- get_legend(plot21)

plot21<-ggplot(data_stations_b, aes(x=RTS, fill=PREG_OUTCOME))+
  geom_bar(stat="count",position="dodge2")+
  scale_x_discrete("Reproductive tract score")+
  scale_fill_manual(values=c("blue", "green", "red"))+
  theme_bw()+
  theme(panel.grid= element_blank(),
        axis.text = element_text(size = font_size, color="black"),
        axis.title = element_text(size = font_size, color="black"),
        legend.position="none")
```

```
grid.arrange(plot16, plot17, plot18, legend, plot19, plot20, plot21,  ncol=4, nrow = 2, 
             layout_matrix = rbind(c(1,2,3,4), c(5,5,6,7)))
```

Figure S4. Percentages of reproductive outcome within different groups of beef heifers categorized by body condition score and reproductive tract score.

```
data_stations_c<-data_stations_b[c("BCS", "RTS" , "PREG_OUTCOME")]
data_stations_d<-data_stations_c %>% group_by(BCS, RTS, PREG_OUTCOME) %>% summarise (n=n()) %>%mutate(freq = n / sum(n))
data_stations_d$percentage<-round(data_stations_d$freq*100,0)
```

```
ggplot(data_stations_d, aes(x=PREG_OUTCOME,  y= percentage, fill=PREG_OUTCOME))+
  geom_bar(stat="identity")+
  scale_fill_manual(name="", values=c("red", "green", "blue"), labels=c("Preg AI", "Preg NS", "Not Preg"))+
  facet_grid(RTS~BCS,switch="y")+
  scale_x_discrete(name="Pregnancy outcome")+
  scale_y_discrete(name="Reproductive tract score")+
  geom_text(aes(label=percentage), vjust=1, color="black", size=3.5)+
  theme_bw()+
  ggtitle("Body condition score")+
  theme(strip.text=element_text(size = 12, color="black"),
        axis.text.x = element_text(size = 12, color="black"),
        axis.text.y = element_blank(),
        axis.title.y = element_text(size = 12, color="black") ,
        axis.title.x = element_text(size = 12, color="black") ,
        axis.ticks.y=element_blank(),
        legend.position="bottom",
        legend.text = element_text(size = 12, color="black"),
        legend.title = element_text(size = 12, color="black"))
```
